# A positive role for PEA3 in HER2-mediated breast tumour progression

**DOI:** 10.1038/sj.bjc.6603427

**Published:** 2006-10-24

**Authors:** E Myers, A D K Hill, G Kelly, E W McDermott, N J O'Higgins, L S Young

**Affiliations:** 1 School of Medicine and Medical Science, Saint Vincent's University Hospital, Dublin, Ireland; 2 Conway Institute, University College Dublin, Dublin, Ireland; 3 School of Mathematical Sciences, Dublin, Ireland

**Keywords:** PEA3, HER2, breast cancer, Ets transcription factor

## Abstract

Overexpression of HER2 is associated with an adverse prognosis in breast cancer. Despite this, the mechanism of its transcriptional regulation remains poorly understood. PEA3, a MAP kinase (MAPK)-activated member of the Ets transcription factor family has been implicated in the transcriptional regulation of HER2. The direction of its modulation remains controversial. We assessed relative levels of PEA3 expression and DNA binding in primary breast cultures derived from patient tumours (*n*=18) in the presence of an activated MAPK pathway using Western blotting and shift analysis. Expression of PEA3 in breast tumours from patients of known HER2 status (*n*=107) was examined by immunohistochemistry. In primary breast cancer cell cultures, growth factors induced interaction between PEA3 and its DNA response element. Upregulation of PEA3 expression in the presence of growth factors associated with HER2 positivity and axillary lymph node metastasis (*P*=0.034 and 0.049, respectively). PEA3 expression in breast cancer tissue associated with reduced disease-free survival (*P*<0.001), Grade III tumours (*P*<0.0001) and axillary lymph node metastasis (*P*=0.026). Co-expression of PEA3 and HER2 significantly associated with rate of recurrence compared to patients who expressed HER2 alone (*P*=0.0039). These data support a positive role for PEA3 in HER2-mediated oncogenesis in breast cancer.

HER2 encodes for a 185 kDa transmembrane glycoprotein receptor which is structurally related to the epidermal growth factor receptor family. Overexpression of HER2 occurs in 20–30% of breast cancer, and is associated with poorly differentiated and highly proliferative tumours (Sorlie *et al*, 2001). These tumours tend to be resistant to endocrine therapies and are associated with a decrease in overall survival (Berns *et al*, 1995; Carlomagno *et al*, 1996). The monoclonal antibody, trastuzumab (herceptin), targets the HER2 receptor and is an effective therapy in recurrent breast cancers that overexpress HER2 (Vogel *et al*, 2002). Despite the prognostic significance of HER2 and the existence of a therapeutic agent, many questions about HER2's role and that of trastuzumab remain. Unfortunately, just as oestrogen receptor (ER) status imperfectly predicts response to endocrine therapy, HER2 expression imperfectly predicts response to trastuzumab (Menard *et al*, 2003). In addition, despite the increased aggressiveness associated with HER2 overexpression, the precise mechanism of HER2 regulation is incompletely understood. Previous studies have implicated growth factor-related transcription factors in HER2 overexpressing breast cancers (Goel and Janknecht, 2003). Ets proteins are a family of mitogen-activated protein kinase (MAPK)-dependant transcription factors. They have been shown to be present in primary human breast cancers and their expression has been associated with disease progression and metastasis (Shepherd *et al*, 2001; Span *et al*, 2002). Ets transcription factors regulate target gene expression by binding to Ets response elements in the promoter region of the relevant target gene. The HER2 upstream regulatory region contains a conserved Ets-binding site and mutation of this sequence reduces transcription of linked reporter genes in several different mammalian cell lines, including breast tumour cell lines (Scott *et al*, 1994). These findings are consistent with the hypothesis that one of the Ets proteins may regulate transcription of the HER2 gene and may account for its increased expression in breast tumour cells. PEA3 (E1AF) is an Ets family member, and previous molecular studies have implicated PEA3 in the regulation of HER2 transcription. Shepherd *et al* (2001) has suggested that PEA3 has the potential to positively regulate HER2 transcription. Conversely, another study suggests that PEA3 represses HER2 promoter-reporter expression in a human ovarian and a human breast carcinoma cell line (Xing *et al*, 2000), and Xia *et al* (2006) have suggested that PEA3 expression does not correlate with HER2 expression in human breast cancer. These conflicting studies prompted us to undertake *ex vivo* studies in human breast tissue and in primary cell cultures derived from patient tumours. We hypothesise that PEA3 expression may be associated either directly or indirectly with HER2 status and that it may be influenced by an activated MAPK pathway. We propose that the direction of these relationships would support either a positive or a negative role for PEA3 in HER2-mediated breast tumorigenesis.

## MATERIALS AND METHODS

### Patient selection

Following ethical approval, 107 breast tumour specimens and six reduction mammoplasties were included in this study. All patients were free of distant metastasis at presentation and were assessed by abdominal ultrasound, chest X-ray and bone scintigraphy before surgery. All patients received chemotherapy and tamoxifen (20 mg day^−1^) for a maximum of 5 years. In those patients who were ER-negative, tamoxifen was prescribed on the basis of the fact that the patients were PR-positive.

### Cell culture stimulations

Following ethical approval, breast tumour specimens were obtained from 18 patients undergoing surgery for removal of a histologically confirmed breast tumour. Breast tumour cell cultures were established and validated as described previously (Myers *et al*, 2004, 2005). In brief, primary tumour epithelial cells were extracted in HBSS without calcium or magnesium (Gibco, Paisley, Scotland) supplemented with 1 *μ*M EDTA and 1 *μ*M DTT for 40 min. Cells were cultured in RPMI containing 5 *μ*g ml^−1^ insulin, 10 *μ*g ml^−1^ transferrin, 30 nM sodium selinate, 10 nM hydrocortisone, 10 nM *β*-estradiol, 10 mM HEPES, 2 mM glutamine, 10% foetal calf serum (w v^−1^) and 5% ultroser G on a growth factor-reduced matirgel matrix (BD Biosciences, San Jose, CA, USA) (60 ng cm^−2^). Examination of primary breast cultures by staining with ethidium bromide and flow cytometric analysis using the phycoerythrin (PE)-labelled pan-leukocyte marker (CD45 RA and RO), confirmed cell viability and epithelial origin of tumour cells (Myers *et al*, 2004). Phenotypically distinct progenitor epithelial cell populations within the mammary epithelium were characterised by flow cytometry using a PE-conjugated mouse anti-human EpCAM (epithelial-specific antigen) antibody and FITC-conjugated mouse anti-human CD227 (MUC1) monoclonal mouse antibody (BD Biosciences). Bipotent progenitors (EpCAM^+^MUC1^−^) which can generate both luminal and myoepithelial cells were found to represent 51.9% of the epithelial cell population, whereas the luminal-restricted progenitor (EpCAM^+^ MUC^+^) were found to represent 48.1%. Cells were incubated in a humidified atmosphere of 5% CO_2_ at 37°C. Experiments were carried out when cells reached 90% confluence. Cells were serum and steroid depleted for 24 h before treatment and then incubated in the presence and absence of bFGF or EGF for 24 h and harvested. Total protein was extracted using lysis buffer (1% Ipegal, 0.5% deoxycholic acid, 0.1% SDS and 1 × PBS) with pefabloc (5 *μ*g ml^−1^). Cell lysates were subsequently normalised for protein content.

### Western blotting

Proteins (100 *μ*g) were resolved on a 12% polyacrylamide gel at 110 V for 120 min and were transferred to a nitrocellulose membrane (250 mA for 60 min). Membranes were incubated for 60 min in blocking buffer (5% non-fat dry milk, 0.1% Tween in PBS) at room temperature and subsequently with primary antibody, mouse anti-human PEA3 (10 *μ*g ml^−1^) (Santa Cruz Biotechnology Inc., Sc-113) in blocking buffer overnight at 4°C. The membranes were washed before incubation with the corresponding horseradish peroxidase secondary antibody (Santa Cruz Biotechnology Inc.) (one in 2000) in blocking buffer for 60 min at room temperature. The membranes were washed and developed with intensified luminescence (Pierce, IL, USA). K-562 cells (Santa Cruz Biotechnology Inc.) were used as a positive control for PEA3.

### Electrophoretic mobility shift assays

Nuclear protein was extracted using a Ner/Per kit according to the manufacturer's instructions (Pierce). For electrophoretic mobility shift assay, 1 *μ*g of nuclear extract was incubated for 30 min in the presence of 20 mM HEPES (pH 7.9), 5 mM MgCl_2_, 20% glycerol, 100 mM KCl, 0.2 mM EDTA, 8% Ficoll, 600 mM KCl, 500 ng *μ*l^−1^ poly dI dC (deoxyinosinic-dexycytidylic) acid, 50 mM DTT and *α*^32^P-dCTP-labelled double-stranded oligonucleotide for Ets, response element. Oligonucleotides were designed to incorporate the native human HER2/ERBB2 (NM_001005852) promoter (−287 to −270) 5′-CATGGCCTAGGGAATTTATCC-3′, with the consensus sequence of Ets-binding elements underlined. For supershift experiments, antibodies against PEA3 were added following the initial incubation, and samples were then incubated for a further 20 min. The samples were electrophoresed through a 5.5% nondenaturing polyacrylamide gel in 0.5 × Tris-borate-EDTA buffer. For competition studies, the reaction was performed as described with 50 × molar excess of unlabelled probe. Supershift negative controls were performed using matched IgG control.

### Immunohistochemistry and immunofluorescence

Five micron thick tissue sections were cut from paraffin-embedded breast tumour tissue blocks and mounted on Superfrost Plus slides (BDH, Poole, UK). Sections were dewaxed, rehydrated and washed in PBS. PEA3 was detected as described previously (Fleming *et al*, 2004). Briefly, sections were blocked in serum for 90 min. Sections were incubated with the primary antibody, mouse anti-human PEA3 (10 *μ*g ml^−1^) and rabbit anti-human Phospho-raf (1 : 50) (Cell signalling, Beverly, MA, USA) for 60 min at room temperature. Subsequently, sections were incubated in the corresponding biotin-labelled secondary antibody (one in 2000) for 30 min, followed by peroxidase-labelled avidin–biotin complex. Sections were developed in 3, 3-diaminobenzidine (DAB) tetrahydrochloride and counterstained with haematoxylin. Negative controls were performed using matched IgG controls (DAKO, Glostrup, Denmark). Immunostained slides were scored for PEA3 and Phospho-raf using the Allred scoring system (Harvey *et al*, 1999). HER2 status was evaluated using the DAKO (Glostrup, Denmark) HercepTest immunocytochemical assay. Scoring was assessed according to the manufacturer's instructions. A score was assigned according to the intensity and pattern of cell membrane staining: 0 to +1=no staining, or staining in <10% of cells; +2=weak to moderate staining in >10% of cells; +3=strong staining in >10% of cells. Sections were examined under a light microscope. Independent observers, without knowledge of prognostic factors, scored slides. Immunofluorescence detection of PEA3 was performed on primary breast cancer tissue. Cells and tissues were prepared as described above. Breast cancer cells and sections were blocked in 1.5% normal serum and then incubated with 20 *μ*g ml^−1^ mouse anti-human PEA3 in 10% human serum for 90 min. Cells and sections were subsequently incubated with Alexa Fluor 546-conjugated secondary antibody (Molecular Probes, Invitrogen, Paisley, UK) for 60 min and were counterstained with DAPI (Sigma-Aldrich, Dorset, UK). Confocal microscopy was performed using a confocal microscope (Zeiss LSM 510 UV META system, Standart Gottingen, Germany) and images were captured using Laser Capture software, Zeiss, Standart Gottingen, Germany.

### Clinicopathological parameters

Variables analysed included tumour grade, axillary nodal status and ER status. A recurrence was defined as any local (chest wall) or systemic recurrence during the follow-up period.

### Statistical analysis

Statistical analysis was carried out using the Fisher's exact test for categorical variables to compare two proportions. Kaplan–Meier estimates of survival functions were computed and the Wilcoxon test was used to compare survival curves. In addition, the Wilcoxon rank sum test was used to compare two medians. Two-sided *P*-values of <0.05 were considered to be statistically significant.

## RESULTS

### Growth factor induction of PEA3 and recruitment to the HER2 promoter in breast cancer cells

**Figure 1:**
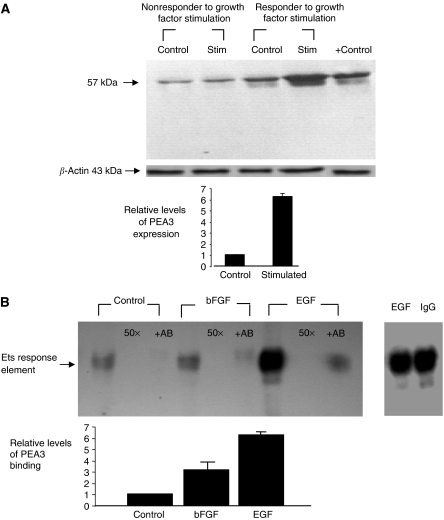
(**A**) Western blot analysis of PEA3 protein levels in primary breast cultures. Illustrative blots of primary tumour response to stimulation with either bFGF (5 ng ml^−1^) or EGF (10 ng ml^−1^). Positive controls, K-562 (+). Relative optical density of PEA3 immunoblots were obtained (Eagle Eye, Stratagene, CA, USA), optical density readings of control values were normalised to 1 and treated groups were expressed as a ratio. Results are expressed as mean±s.e.m. (*n*=18). (**B**) Electrophoretic mobility shift analysis of nuclear extracts primary breast cancer cultures. Nuclear protein extracts from primary breast cancer cells in the presence and absence of bFGF and EGF were compared for increased binding to an *α*^32^P-dCTP labelled Ets response element. DNA protein interactions were assayed in the presence of 50 × molar excess of homologous oligonucleotide. Nuclear protein extracts were pre-incubated in the presence of anti-PEA3. Negative controls were performed using matched IgG. Relative optical densities of PEA3 immunoblots were performed. Optical density readings of control values were normalised to 1 and treated groups were expressed as a ratio. Results are expressed as mean±s.e.m. (*n*=3).

The ability of growth factors bFGF and EGF to induce PEA3 expression in primary breast cancer cells derived from patient tumours was investigated by Western blotting. The transcription factor was expressed in a proportion of tumours (14 out of 18 tumours). Of tumours found positive for PEA3, increased expression was found in a subset of tumours in response to growth factor treatment (Figure 1A).

To determine the ability of PEA3 to bind to the Ets response element in the presence of growth factors, bFGF and EGF, gel shift assays were performed. Using oligonucleotide sequences, which are specific for the HER2 promoter, the ability of nuclear extracts from non-treated, primary breast cancer cell cultures to bind to the DNA response element was compared to cells treated with bFGF and EGF (Figure 1B). PEA3 response element binding was induced in the presence of both bFGF and in particular EGF in comparison to control. An immuno-depletion induced by pre-incubation of the nuclear extracts with anti- PEA3 established that PEA3 was present at the protein–DNA complex.

### Localisation of PEA3 and Phospho-raf in human breast cancer

**Figure 2:**
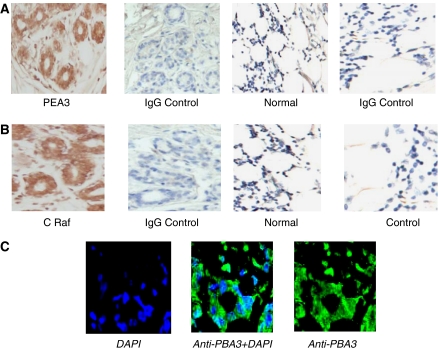
(**A**) Immunohistochemical localisation of PEA3 (× 200) counterstained with haematoxylin and matched IgG-negative controls in human breast cancer tissue. (**B**) Immunohistochemical localisation of Phospho-raf (× 200) counterstained with haematoxylin and matched IgG-negative controls in human breast cancer tissue. (**C**) Immunofluoresence localisation of PEA3 (green) in human breast cancer tissue (× 630) counterstained with DAPI (blue).

The transcription factor PEA3 was localised within paraffin-embedded human breast tissue using immunohistochemistry. PEA3 was detected predominantly within the nuclei of invasive tumour epithelial cells, and to a lesser extent within the cytosol (Figure 2A). PEA3 protein was not detected in the normal surrounding breast tissue nor in the reduction mammoplasties, which were used as controls. This predominant nuclear localisation was confirmed by immunofluorescence (Figure 2C). PEA3 was found to be expressed in 47% of breast tumour patients. As reported previously, expression of Phospho-raf was found within the nuclei of tumour epithelial cells with scant cytoplasmic staining (Myers *et al*, 2005) (Figure 2B).

### Associations between the expression of PEA3 and clinical variables/growth factor markers

**Figure 3:**
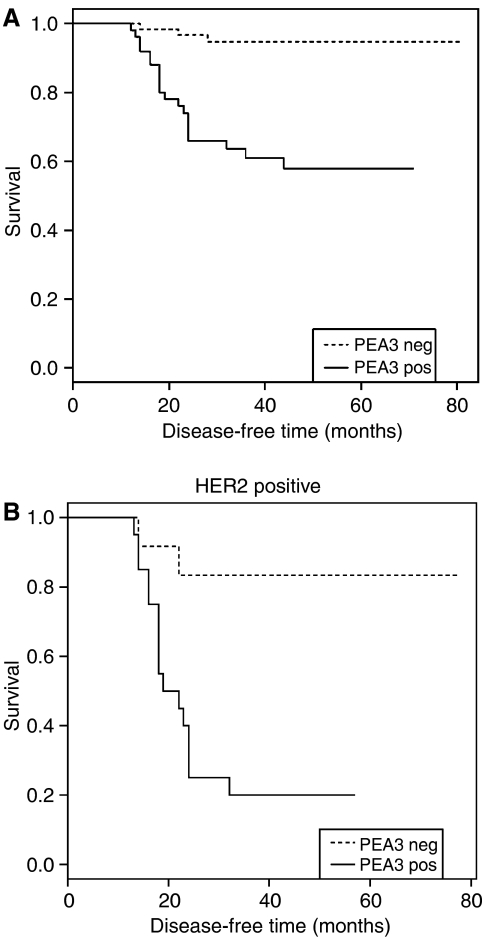
(**A**) Kaplan–Meier estimates of disease-free survival (DFS) (*n*=107) according to PEA3 expression. (**B**) DFS according to PEA3 expression in HER2-positive breast tumour patients (*n*=32).

Associations between the qualitative expression of PEA3 and clinicopathological parameters were examined. Expression of the transcription factor, PEA3, was found to associate with both tumour grade and axillary lymph node positivity (*P*<0.0001 and 0.026, respectively). A significant association was found between disease recurrence and expression of PEA3 (*P*<0.0001). No relationship was detected between the transcription factor and ER status (Table 1). From Kaplan–Meier estimates of survival, PEA3 protein was found to significantly associate with time to disease recurrence (*P*<0.001, *n*=107) (Figure 3A).

In line with previous observations (Fleming *et al*, 2004), a significant association was detected between PEA3 expression and that of HER2 (*P*=0.0369) (Table 1). The ability of breast cancer cells derived from patient tumours to regulate PEA3 protein expression in the presence of growth factors was related to clinicopathological parameters. Upregulation of PEA3 was detected in 66% (12 out of 18 tumours) of tumours. Relative increases in PEA3 protein expression are given in Table 2. Growth factor induction of PEA3 expression was found to significantly associate with axillary lymph node positivity and expression of HER2 (*P*=0.049 and *P*=0.034, respectively). Co-expression of HER2 and PEA3 significantly associated with increased rate of recurrence, compared to patients who expressed HER2, but not PEA3 (*P*=0.0039, *n*=32) (Figure 3B). In order to determine the association between PEA3 status and an activated MAPK pathway, expression of the MAPK protein Phospho-raf was examined. Phospho-raf expression significantly associated with PEA3 expression (*P*<0.0001) (Table 1).

## DISCUSSION

The HER2 (erbB2) gene encodes a 185-kDa transmembrane receptor with tyrosine kinase activity and acts through intracellular signal transduction pathways to alter gene expression. HER2 overexpression is associated with aggressive breast cancers and an adverse prognosis. Trastuzumab (herceptin) is a monoclonal antibody which targets HER2 and combination chemotherapy with trastuzumab is now a standard first-line treatment for women with advanced HER2 overexpressing breast cancer (Vogel *et al*, 2002). Recent data support trastuzumab's role in earlier stages of disease (Romond *et al*, 2005). Despite these advances, a number of questions remain regarding the exact role of HER2 in breast tumour progression including the exact mechanism of HER2 transcriptional regulation. The Ets family of transcription factors play a well-documented role in breast tumour progression and have been shown to contribute to the transcriptional regulation of HER2 (Shepherd *et al*, 2001; Span *et al*, 2002). We have previously demonstrated that the growth factor EGF induces HER2 protein expression in primary breast cultures derived from patient tumours, thus implicating an activated MAPK pathway in HER2 transcriptional regulation (Myers *et al*, 2005). To date, assessing endogenous PEA3 protein expression in human breast cancer cell lines has proved difficult. Here, we demonstrate an upregulation in PEA3 protein expression and DNA binding within the HER2 promoter in the presence of an activated MAPK pathway and describe a relationship between regulation of PEA3 expression in response to growth factor stimulation and HER2 positivity in primary breast tumour cultures. In this study, we describe associations between co-expression of PEA3 and HER2 and time to disease recurrence.

Ets family members can be divided into 13 subgroups based on the sequence similarity of their Ets domains. PEA3 is a founding member of a subfamily of class I Ets transcription factors that also includes ER81 and ERM (Xin *et al*, 1992). The Ets family of transcription factors contribute to the transcriptional regulation of HER2 (Shepherd *et al*, 2001). The exact role of PEA3 in HER2 transcriptional regulation remains controversial with conflicting reports as to the direction of its modulation (Xing *et al*, 2000; Shepherd *et al*, 2001). Ets transcription factors are known effectors of the MAPK pathway, (O'Hagan *et al*, 1996), and class I family members, in particular, are targets for phosphorylation in response to stimulation. Phosphorylation of Ets sub-family members, Ets-1 and Ets-2, by MAPK-dependent pathways leads to persistent expression of tumour-related target genes including proteases such as uPA and MMPs (Yang *et al*, 1996; Sharrocks *et al*, 1997). *In vitro* studies provide evidence that PEA3 can be activated through phosphorylation by MAPK pathway, both through the extracellular signal-related kinase and the c-Jun N-terminal kinase stress-activated protein kinase (Wasylyk *et al*, 1997). Phosphorylation via an activated MAPK pathway also represents a potential mechanism of activation of PEA3-DNA-binding (O'Hagan *et al*, 1996). We found that both the growth factors, bFGF and EGF, induced interaction between PEA3 and its response element, within the promoter region of HER2. In this study, growth factors, bFGF and EGF upregulated the protein expression of PEA3 in human primary tumour cell cultures. Furthermore, response to growth factor stimulation in primary breast tumour cultures associated with axillary lymph node positivity and HER2 overexpression supporting a positive role for PEA3 in breast cancer progression.

A previous study by Kinoshita *et al* (2002) observed PEA3 protein to be expressed exclusively in tumour tissue. We have previously correlated PEA3 protein expression with that of HER2 in a limited cohort of breast cancer patients (Fleming *et al*, 2004). In this study, we have examined the expression of PEA3 in relation to established clinical parameters of breast cancer. We found a positive association between PEA3 protein expression and tumour grade and axillary lymph node positivity, known poor prognostic indicators in breast cancer. In addition, PEA3 expression correlated with time to disease recurrence. Co-expression of PEA3 and HER2 significantly increased the rate of disease recurrence supporting a positive role for PEA3 in HER2-mediated oncogenesis.

Associations between co-expression of PEA3 and HER2 and reduced disease-free survival and between upregulation of PEA3 protein expression, in primary cultures, and both axillary lymph node positivity and HER2 overexpression are suggestive of a positive role for PEA3 in HER2-mediated breast tumour progression.

## Figures and Tables

**Table 1:** Comparison of PEA3 expression with clinicopathological parameters and growth factor markers

	**Total**	**PEA3-positive (%)**	**PEA3-negative (%)**	***P*-value**
No. of patients	107	50 (47%)	57 (53%)	—
Mean age		47.94	51.90	0.0776
Tumour size (mm)		3.17	2.72	0.0198
				
*Grade*				
Grade 3	55	37 (67%)	18 (33%)	
Non-grade 3	52	13 (25%)	39 (75%)	<0.0001
				
*Axillary lymph node status*				
Node-positive	69	38 (55%)	31 (45%)	
Node-negative	38	12 (32%)	26 (68%)	0.0260
				
*ER status*				
Positive	84	40 (48%)	44 (52%)	
Negative	23	10 (43%)	13 (57%	0.8154
				
*HER2 status*				
Positive	32	20 (63%)	12 (37%)	
Negative	75	30 (40%)	45 (60%)	0.0369
				
*Recurrence*				
Positive	23	20 (87%)	3 (13%)	
Negative	84	30 (36%)	54 (64%)	<0.0001
				
*Phospho-raf*				
Positive	37	31 (84%)	6 (16%)	<0.0001
Negative	70	19 (27%)	51 (73%)	

ER=oestrogen receptor.

Continuous variables were analysed using the two-sample *t*-test. Nominal variables were analysed using Fisher's exact test.

**Table 2:** Relative levels of protein expression of PEA3 in primary breast tumour cell cultures in the presence of growth factors, *n*=18

	**PEA3_stimulated_ – PEA3_control_**	***P*-value**
No. of positive patients	14/18	
		
*HER2 status (median and range)*		
Positive	534 (200–860)	0.034
Negative	124 (0–340)	
		
*Axillary status (median and range)*		
Positive	338 (160–660)	0.049
Negative	80 (0–210)	

Relative optical density of PEA3 immunoblots both under control conditions and following stimulation with growth factors were obtained. Optical density readings were normalised to 1 and treated groups were expressed as a ratio. Data expressed as median and range, comparisons analysed using Wilcoxon rank sum test. These data are also represented graphically in the form of a histogram.
